# Radial Growth of Qilian Juniper on the Northeast Tibetan Plateau and Potential Climate Associations

**DOI:** 10.1371/journal.pone.0079362

**Published:** 2013-11-14

**Authors:** Chun Qin, Bao Yang, Thomas M. Melvin, Zexin Fan, Yan Zhao, Keith R. Briffa

**Affiliations:** 1 Key Laboratory of Desert and Desertification, Cold and Arid Regions Environmental and Engineering Research Institute, Chinese Academy of Sciences, Lanzhou, China; 2 Climatic Research Unit, School of Environmental Sciences, University of East Anglia, Norwich, United Kingdom; 3 Key Laboratory of Tropical Forest Ecology, Xishuangbanna Tropical Botanical Garden, Chinese Academy of Sciences, Kunming, China; 4 Institute of Geographic Sciences and Natural Resources Research, Chinese Academy of Sciences, Beijing, China; Universidade Federal do Rio de Janeiro, Brazil

## Abstract

There is controversy regarding the limiting climatic factor for tree radial growth at the alpine treeline on the northeastern Tibetan Plateau. In this study, we collected 594 increment cores from 331 trees, grouped within four altitude belts spanning the range 3550 to 4020 m.a.s.l. on a single hillside. We have developed four equivalent ring-width chronologies and shown that there are no significant differences in their growth-climate responses during 1956 to 2011 or in their longer-term growth patterns during the period AD 1110–2011. The main climate influence on radial growth is shown to be precipitation variability. Missing ring analysis shows that tree radial growth at the uppermost treeline location is more sensitive to climate variation than that at other elevations, and poor tree radial growth is particularly linked to the occurrence of serious drought events. Hence water limitation, rather than temperature stress, plays the pivotal role in controlling the radial growth of Sabina przewalskii Kom. at the treeline in this region. This finding contradicts any generalisation that tree-ring chronologies from high-elevation treeline environments are mostly indicators of temperature changes.

## Introduction

Due to its exposure to extremely harsh environmental conditions, such as low air temperatures and severe water shortage, tree growth near the alpine treeline is potentially extremely sensitive to climate change [1]. Consequently, such environments have received increasing attention from dendroclimatologists. Based on the principle of limiting factors in dendroclimatology, many dendroclimatologists consider that the variability of annual tree growth at the lower treeline is likely controlled by variability in precipitation, while at the upper treeline it is more closely controlled by the variability of temperature, especially at high latitudes, or at high elevations in the generally humid Alps and in some semi-arid areas [2]–[6]. However, uniform growth pattern (where the tree growth is controlled by a common climate signal at upper and lower treelines) has also been found in the arid regions of western central Asia [7], in the northeastern region of the arid central Tianshan Mountains [8], in a subtropical monsoonal region of South America [9] and in a low latitude humid region of North America [10], [11]. Therefore, further researches are required to clarify the association between elevation and climate influences on tree growth in the regions of various climate types.

The Northeast Tibetan Plateau, located at the convergence of the westerly and easterly summer monsoons, is highly sensitive to climate change. The altitude of the terrain in this region varies from 2000 m to 5050 m above sea level (a.s.l.). Qilian juniper and Qinghai spruce (*Picea crassifolia Kom*.) are endemic and widespread dominant species, on south-facing slopes and north-facing slopes, respectively, in the alpine forest of the northeast Tibetan Plateau. Both species are generally considered as good candidates for obtaining effective paleoclimate proxies [12]–[19]. There are a number of previous studies of tree growth and climate variability in the treeline ecotone in this area [20]–[29]. Some researchers consider that temperature is the main growth-limiting factor in the upper treeline ecotone: for example in the eastern Qilian Mountains [27], in the eastern mountainous regions of the Qaidam Basin [23], [28], in the semi-humid climates of the southeastern [20] and eastern [21] of the Tibetan Plateau. Other researchers have found that the growth responses of tree-line forests to climate variability show different patterns, associated with different climate conditions in the Anyemaqen Mountains [24] and the southern Tibetan Plateau [29], and have shown that short-term growth-climate relationships are unstable in the Qilian Mountains [26]. In summary, the effect of climate change on the alpine treeline in our study area remains complex and a matter of debate. The focus of attention in this study concerns the main limiting factor for tree growth in the treeline ecotone: is it temperature, precipitation, or both? Thus, we start from the plant physiological/ecological viewpoint, exploring altitude and age-dependent characteristics of tree growth in this species, and combine this with dendroclimatology, exploring the statistical association between tree radial growth time series and climate data records to establish a case study on the northeastern Tibetan plateau, China. This information will hopefully provide new insights that will improve our understanding of the influence of climate variability on the radial growth of Qilian juniper (*Sabina przewalskii Kom*.) at the alpine treeline in the semiarid region.

## Materials and Methods

All necessary permits were obtained for the described field studies from the Administration of Delingha Forestry.

### Study area

The study area is located in the Zongwulong Mountains, on the northeast Tibetan Plateau. The altitude of the study area varies between 3200 m and 5030 m a.s.l, with an average altitude of 4000 m. There is perennial snow cover above 4500 m.a.s.l. Qilian juniper (on south-facing slopes), Qinghai spruce (on north-facing slopes) and more prostrate shrub growth are found below 4100 m.a.s.l.. Qilian juniper is mostly distributed in the upper regions of the mountains, while the tops are mostly characterized by rock debris and rocky outcrops. The mountains are mainly composed of middle-Jurassic sandstone, conglomerate and limestone. Alpine forest gray–brown soils, subalpine shrub meadow soils, alpine meadow soils, and alpine chestnut soils are typical in this area.

**Figure 1:**
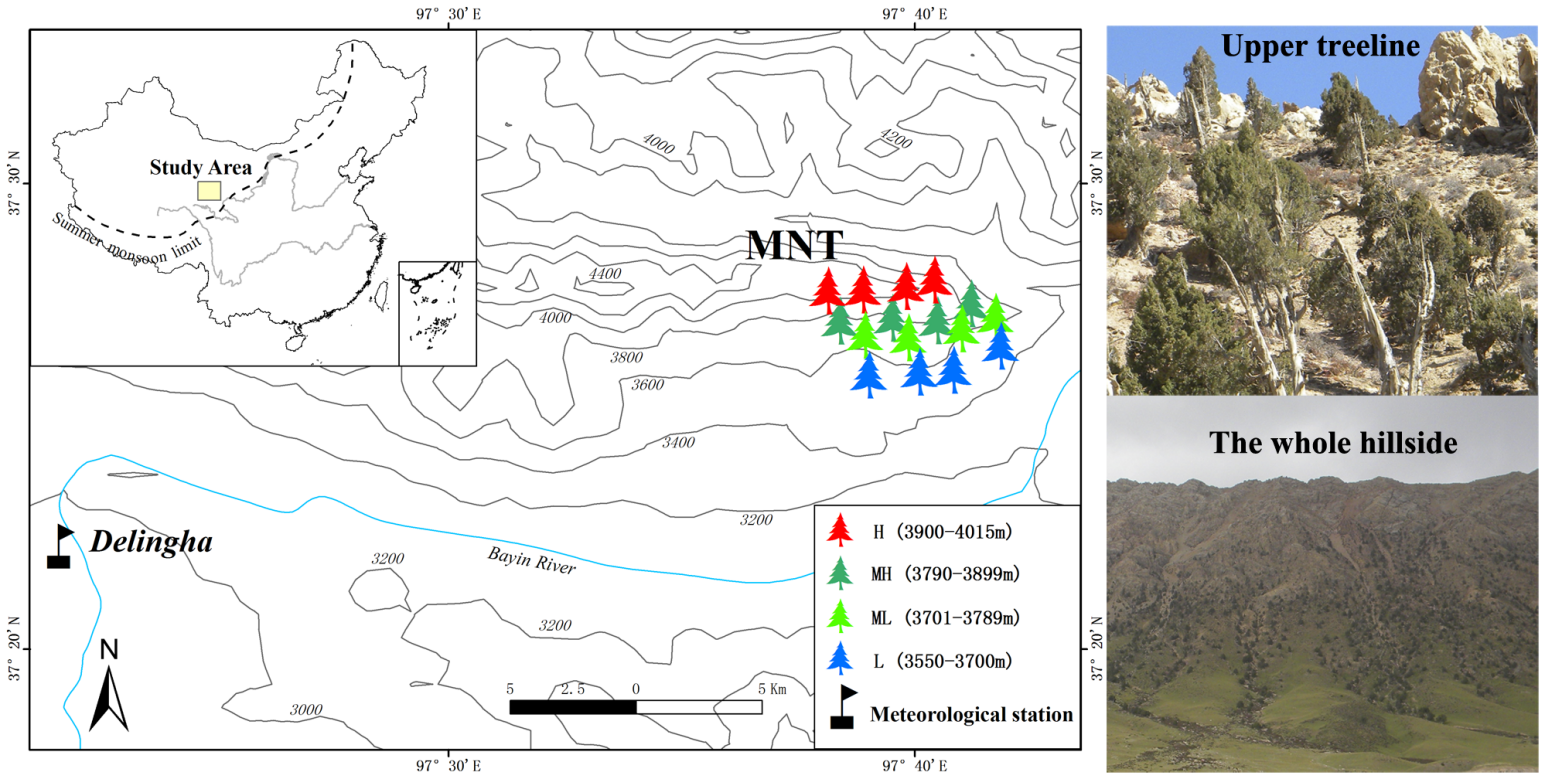
Locations of the tree-ring sampling sites and the meteorological station on the northeast Tibetan Plateau (black flag: Delingha meteorological station; red tree, olive green tree, green tree and blue tree indicates the ring-width collection areas at different elevations: H (3900 m–4015 m), MH (3790 m–3899 m), ML (3701 m–3789 m), and L (3550 m–3700 m); two photographs were taken of the upper treeline and the whole hillside, respectively).

**Figure 2:**
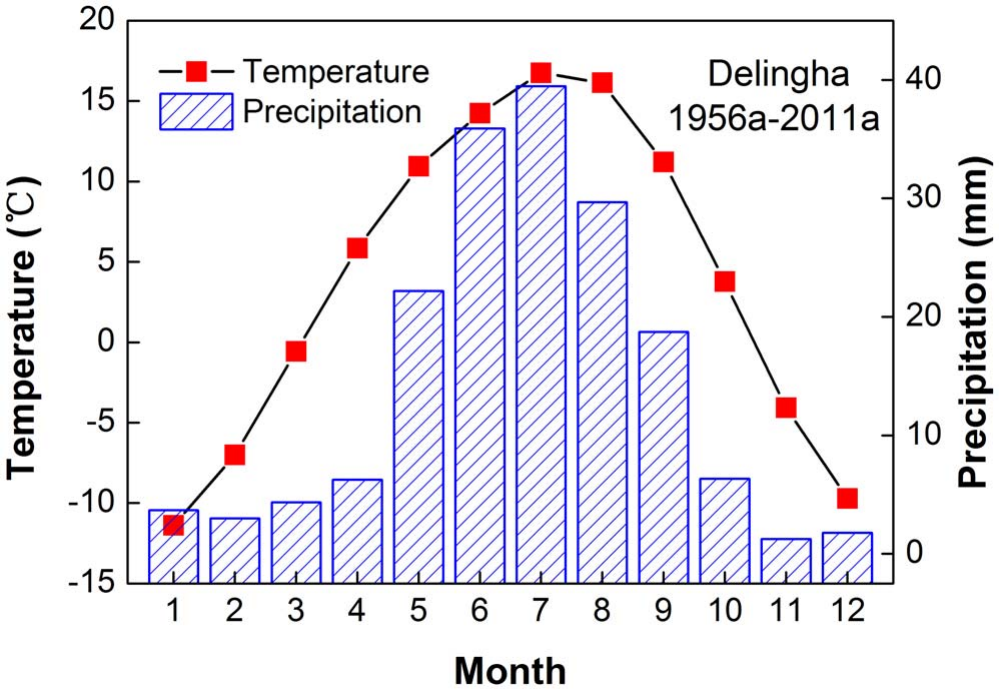
Climate diagrams of temperature and precipitation at Delingha meteorological station for the 1956–2011.

The individual sampling sites are located in a sampling region called Manitu (MNT, 97.68°E, 37.46°N, Figure 1), on a south-facing (sunny) slope in the middle of the Zongwulong Mountains. The slope angle ranges from 15° to 65°. The alpine forest is pure Qilian juniper forest. The closest available meteorological records are from Delingha national weather station (37.37°N, 97.37°E, 2982.4 m.a.s.l.), which is 41 kilometers from MNT and between 500 m and 1000 m below the study sites. The mean, maximum and minimum annual temperatures at Delingha were 3.84°C, 11.38°C and −2.55°C in the period 1956–2011, respectively. Mean winter (from previous December to current January) and summer (from June to July) temperatures were −9.4°C and 15.7°C. The average annual precipitation was 173 mm, with 73.7% of the annual precipitation falling between May and August. The average relative humidity was 38.8%. In summary, our study area represents a typical mid-latitude arid to semi-arid alpine region (Figure 2).

### Tree-ring width data

All the tree-ring width (TRW) samples were collected at breast height with an increment borer, on a single hillside. After air drying, the core surfaces were prepared with razor blades and the surface contrast was enhanced with chalk. Ring widths were recorded with a LINTAB II measuring system at a resolution of 0.01 mm, and all cores were cross-dated by visual growth pattern matching, skeleton plotting and statistical tests available in the software package TSAP [30], [31]. Cores displaying poor correlation with the master series were removed. Finally, 594 increment cores from 331 trees were retained. Detailed information on sampling sites and the TRW data at different elevations are listed in Table 1. Based on the elevation range and the characteristics of the sample distribution, we separated the data into four different altitude belts stretching from the lower tree line to the upper tree line, while ensuring that sufficient samples were available in each altitude belt. These four ranges were as follows: High (H: 3900–4015 m.a.s.l.), Middle High (MH: 3790–3899 m.a.s.l.), Middle Low (ML: 3701–3789 m.a.s.l.), and Low (L: 3550–3700 m.a.s.l.).

**Table 1:** Characteristics of raw (measured) tree-ring data and of the standard chronologies for each of four (High, Middle High, Middle Low and Low) elevations and for the overall composite chronology.

Site code	Altitude (m)	Slope(°)	Core/Tree	Time span(AD)	ML	Max/Mean/Min TRW (mm)
H	3900–4015/3965	40–65	165/93(45)	955–2011	452	0.667/0.331/0.127
MH	3790–3899/3818	40–65	147/85(37)	1005–2011	447	0.671/0.352/0.131
ML	3701–3789/3760	35–60	165/91(54)	940–2011	436	0.874/0.394/0.205
L	3550–3700/3603	15–45	117/62(31)	1110–2011	369	1.331/0.513/0.169
ALL	-	-	594/331(167)	940–2011	430	1.331/0.39/0.127

Site code	MS	AC(1)	R/R1/R2*	PC1*(%)	SNR*	EPS*
H	0.33/0.22	0.70/0.33	0.57/0.89/0.57	44.9/59.4	26/50	0.98
MH	0.36/0.27	0.70/0.50	0.57/0.92/0.56	44.9/61.1	17/36	0.97
ML	0.32/0.25	0.73/0.54	0.4/0.78/0.39	30.8/42.5	14/28	0.96
L	0.34/0.25	0.74/0.62	0.52/0.87/0.49	43.4/56.8	6/11.8	0.92
ALL	0.34/0.25	0.72/0.49	0.48/0.85/0.47	37.8/66.5	42/54	0.99

Altitude: elevation range/median elevation; Slope: topographic slope angle; Core/Tree: number of cores and trees collected at this elevation, the value in round brackets is the number of trees for which pith offsets are available; Time span: the period when the EPS value is greater than 0.85,which means the time series is robust. ML: mean segment length; Max/Mean/Min TRW: maximum, mean and minimum tree-ring width; MS: mean sensitivity of raw ring data and standard chronology; AC1: first-order autocorrelation of raw ring data and standard chronology; R/R1/R2: mean inter-series/within-tree/between-tree correlation coefficient; PC1: percent variance explained by the first principal component of raw ring data and standard chronology; SNR: signal-to-noise ratio of raw ring data and standard chronology; EPS: expressed population signal, calculated for ARSTAN standard chronologies for 30-year intervals with 15-year overlaps.

* Calculated for the common period 1750–2000 (1700–2000 for the composite chronology).

### Climate data

Monthly mean/maximum/minimum temperature, total precipitation and mean relatively humidity data were computed from daily records for Delingha station obtained from the China Meteorological Data Sharing Service System (http://cdc.cma.gov.cn/). The instrumental records cover 1956–2011. The nearest Palmer Drought Severity Index (PDSI) gridpoint data (1850–2010, for a 2.5°×2.5° grid, centered on 36.25°N, 98.75°E) were extracted from the self-calibrated PDSI data set, in this case calculated using Penman-Monteith Potential Evapotranspiration (updated to 2010) [32]. For consistency with other climate data, we used the PDSI data for the period 1956 to 2010.

### Radial tree growth analysis

Measured tree-ring radial growth series comprise age-related trends, climate-related growth variations, the effects of disturbance from within and outside the forest community, and residual variability associated with random disturbance effects [33], [34]. Because the canopy density of Qilian juniper is less than 0.3, we adopted the simplifying assumption that trees were growing in a relatively competition-free, open-canopy forest. We assumed a situation where an idealized series of radial growth increment measurements contains only age-related effects manifested as a long-term radial growth trend with climate-related variations superimposed. We also note that “elevation is a site factor affecting mean annual precipitation and temperature regimes” [3]. Tree-ring width (TRW) variability is therefore attributable to a combination of altitude-related variability (including climate) and age-related trends, specifically:
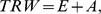
where E is the elevation-related variability, and A represent the age-related trends.

### Chronology analysis

To investigate the climate-related variability, ‘growth trends’ were firstly removed from the series of ring width measurements. All processing was carried out using the ARSTAN program [34]. The raw TRW series were ‘standardized’ to remove biological growth trends and other low-frequency variations considered attributable to stand dynamics. Before standardization, the variance of each series was stabilized using a data-adaptive power transformation based on the local mean and standard deviation [35]. In order to remove age-related biological trends, a modified negative exponential curve was fitted to each raw series. The tree-ring indices were obtained by calculating residuals between raw measurements and fitted values. All detrended index series were then averaged using the biweight robust mean (rather than the arithmetic mean) in order to reduce the influence of outliers [36]. Four TRW chronologies were developed in this way, each representing tree growth over time, on relatively short-to-medium timescales (years to centuries) in each of the four altitude belts.

Standard (STD) and residual (RES) are two types of tree chronology produced by ARSTAN, each representing complimentary information useful for climate reconstruction. However, there are essential differences in terms of trend persistence. Due to the removal of low-order persistence, the RES chronology only represents relatively ‘high-frequency’ information, while STD contains more low-frequency information originating from persistent variance in single trees. To investigate growth-climate relationships, we employed both types of TRW chronology. The relevant data will be deposited in PANGAEA (http://www.pangaea.de/).

### Growth-climate response analysis

Due to the biological persistence in TRW series, TRW series often represent an effectively smoothed response to climate variability registered on a year-to-year basis. To explore the statistical associations between different monthly-mean climate factors and ring-width indices, we selected the period encompassing the previous May to the current October as the “potential effective climatic window”. Individual monthly climate–growth relationships were analyzed using the software DENDROCLIM 2002 [37]. Based on the results, the optimum seasonal assemblies were selected for the growth-climate response analysis.

Unfortunately, there are only 56 years of instrumental climate records available, which is too short to identify the natural relationships between tree radial growth and climate change trends with strong confidence. Meanwhile, TRW index data show 1956–2011 to be a period of relatively high growth (mean value 1.11) relative to the long-term index average (the mean value is 0.99 from 1110 to 2011), limiting our ability to assess whether there are existing significant altitude-related impacts of climate on radial growth in our study area. We also note that the chronology construction approaches used here (i.e. the STD and RES chronologies from ARSTAN – see earlier) may not retain all of the long-timescale climate-related information. We selected six ‘typical’ 50-year periods (comprising three high-growth periods and three relatively low-growth periods): 1204–1253 (high); 1254–1303 (low), 1435–1484 (low), 1550–1599 (high), 1684–1733 (low), and 1961–2010 (high). We treated each TRW index series as a 50-dimensional vector and computed the Euclidean distance between each individual vector and the mean 50-dimensional vector (the mean value of each series on each year), using cluster analysis, to measure the difference between each single TRW index at different elevations.

The equation for Euclidean distance is 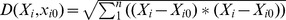, where 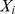 is the 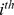-dimension value of each vector and 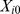 is the mean value of all the 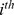-dimension values. Here, *n* = 50 years.

### Extreme climate events analysis

Missing rings are an excellent indicator of likely extreme climate events when the meteorological conditions are assumed to be beyond the physiological limit for tree growth. In general, we would expect the frequency of missing rings to be associated mostly with the occurrence of extreme drought-related events in this semi-arid area. We wished to investigate the nature of the relationship between missing ring and climate variability and whether there is evidence of significant altitude-related effects on this relationship. In order to answer these questions, we computed the temporal variability of the relative missing ring ratio in each altitude belt. To reduce sample depth-related effects (i.e. differing replication), homogenization of sample depth was applied prior to computing the relative missing ring ratio. The Kruskal-Wallis H test, a type of K independent samples nonparametric test, was applied to test the difference.

## Results

### Characteristics of radial growth

**Figure 3:**
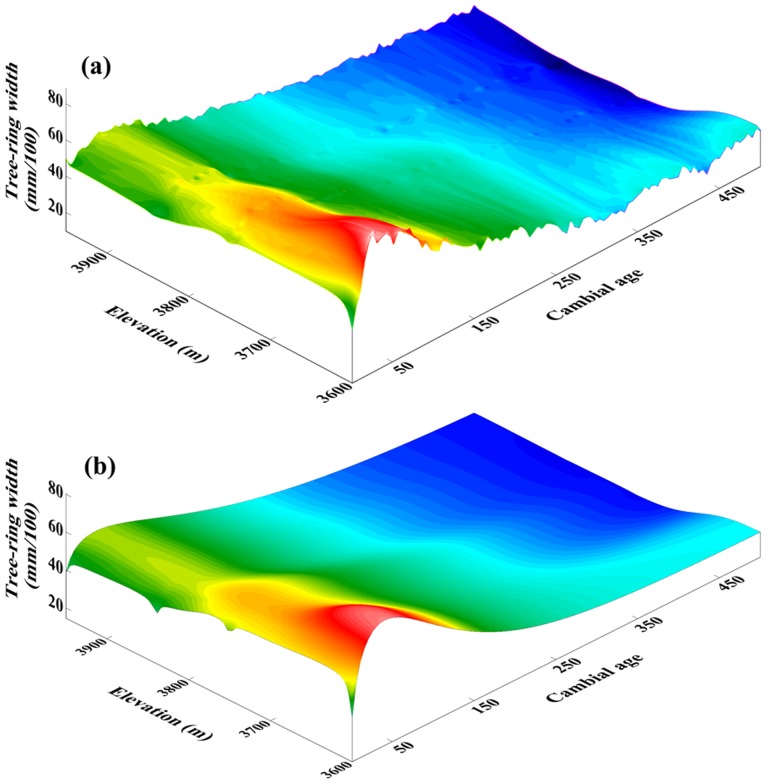
Trend surface for cambial age, elevation (m), tree-ring width (mm/100) (a) and radial ring-width growth trends (mm/100) (b) at MNT, Delingha, China. The surface is interpolated from four original unsmoothed curves of radial ring width (see Figure 5) using inverse distance weighting.

In order to establish a robust picture of the physiological-ecological trend curve, we examined tree cambial age only up to 500 years, when the sample depths were 19 trees (for the high elevation group: H), 14 trees (MH), 22 trees (ML), and 13 trees (L), respectively. Figure 3 clearly shows that radial growth of Qilian juniper has a distinct age-related trend, regardless of whether the trees grow at high or low altitude, starting with a very rapid initial growth rate peaking at ages 25–45. Subsequently, the growth rate gradually decreases with increasing age. This pattern is consistent with our previous research on the same species [38] in the upstream Heihe river region (38°09′N, 99°57′E).

**Figure 4:**
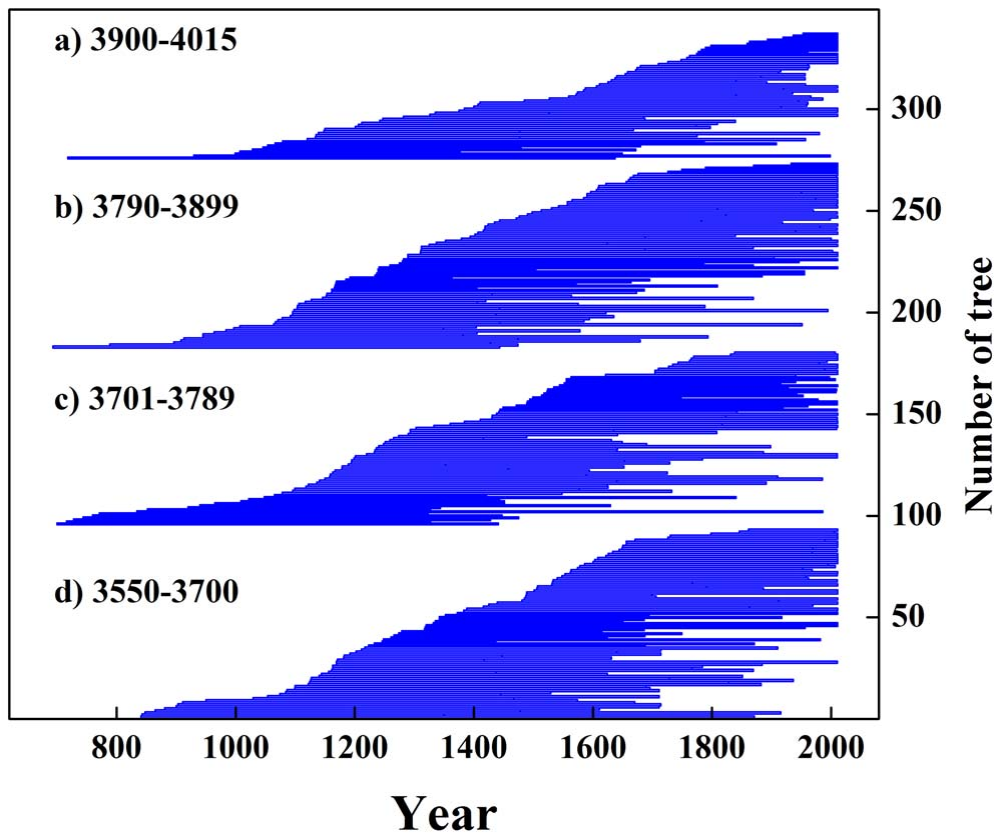
Comparison of sample depths over time for the four elevation zones.

**Figure 5:**
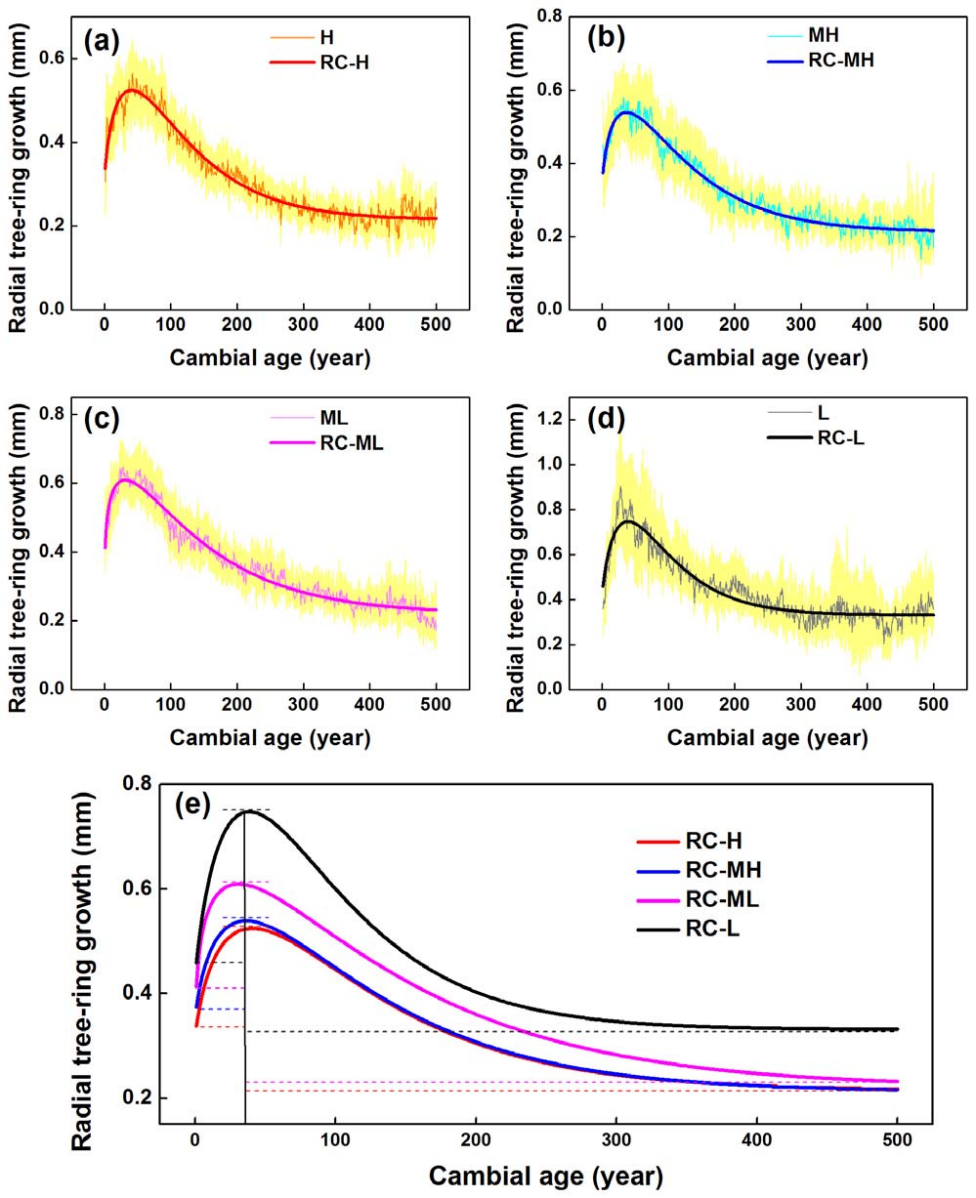
Characteristics of the radial tree-ring biological growth trends at different elevations: (a) H (3900 m–4015 m), (b) MH (3790 m–3899 m), (c) ML (3701 m–3789 m), and (d) L (3550 m–3700 m), based on Hugershoff functions fitted to the means measurement data. The thin line is a mean curve for radial tree-ring biological growth at different elevations. The thick line is a curve fitted to the Hugershoff function. The 95% confidence interval is shown by light yellow shading.

Meanwhile, altitude-related variations are evident during the different lifestages of Qilian juniper. Due to the similar distribution of young and old trees within each altitude belt (Figure 4), the four ring-width groups display similar age-related “disturbances” in the age-related expectation of ring-width as a function of tree age [39]. In general, TRW decreases with increasing elevation (Figure 3, Table 1), regardless of whether a tree is old or young. However, the amplitude of the average ‘cycle’ of changing ring width throughout the lifespan of trees is markedly lower (from 0.29 mm to 0.16 mm in the beginning 50 years, and from 0.42 mm to 0.30 mm over the whole 500 years) with increasing altitude (Figure 5). By 500 years the annual growth rate has reduced from 0.33 mm to 0.21 mm. The age of maximum ring width attainment is similar at each of the four elevations (specifically, H: 41, MH: 37, ML: 31, L: 39; years of age). The radial ring-width decline with age after trees reach about 50 years old is steeper at lower elevation sites. This physiological phenomenon is directly related to the tree growth rate (i.e. increase of diameter with age): lower elevation trees exhibit a faster growth rate. Such differences in radial growth rates at the same biological age are the result of local environmental effects of temperature and precipitation due to different elevations.

Mean series length (i.e. the age of sample trees) increases with increasing elevation, which likely reflects more human disturbance (e.g., logging, grazing, man-made fire) at the lower boundary and less disturbance at the upper treeline. It is worth noting that the confidence associated with fitting trend surfaces in the 350–500 years range is low, because the minimum of the mean series length in four elevations (Table 1) is nearly 350 years; this necessarily restricts our discussion of the biological age 350–500 due to the effective “high-pass-filtering effect” on the variance spectra of tree indices and on the chronology series imposed by the standardization methods used here. This is the so-called “segment length effect” described in Cook et al. [40] and Esper et al. [41].

**Figure 6:**
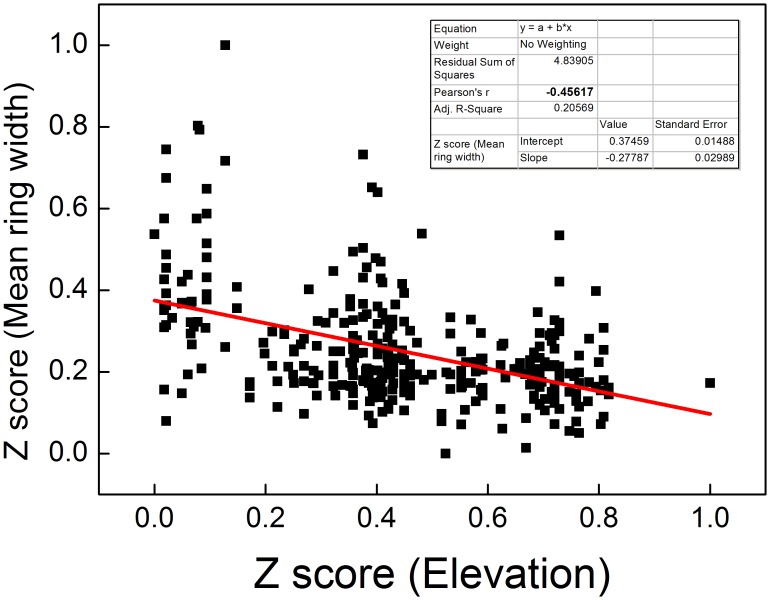
Scatter diagram showing relationships between the elevation and mean ring width after Z score transformation using 265 trees. The Pearson's correlation coefficient is −0.29, which is significant at the p = 0.01 level.

In order to quantify the relationships between TRW and elevation, we removed the fast growth period (about 300 years) at the beginning of each series for each tree and computed the Z-Score of TRW and elevation of 265 trees, and plotted a scatter diagram (Figure 6). The results show that TRW and elevation have a significant negative correlation (*R = −0.28, N = 265*) at the 0.01 level. Therefore, elevation significantly affects radial (physiological) tree growth of Qilian juniper species. These results provide a basis for the priority of growth rate classification or altitude classification pretreatment of TRW over the tree-ring growth trend removal when exploring the use of Regional Curve Standardisation.

### Characteristics of the tree ring index

**Figure 7:**
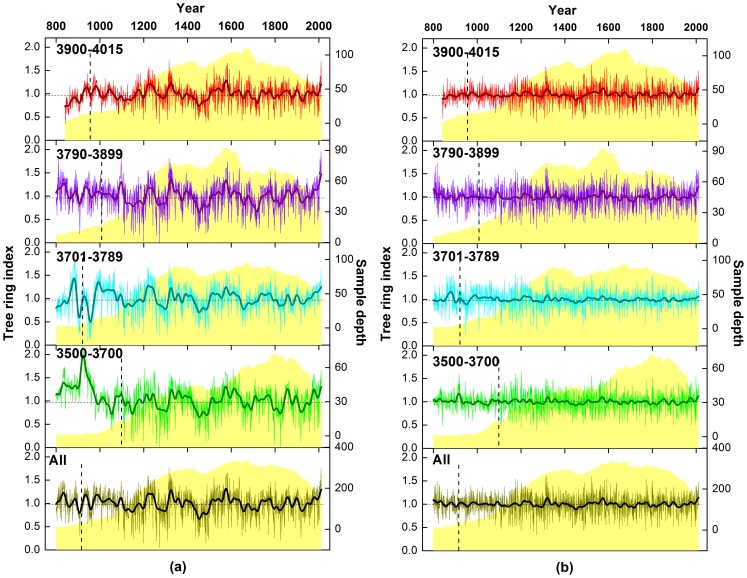
Four standardized chronologies (a), four residual chronologies (b) and the composite chronology (All) and their sample depth at different elevations from MNT. The regional chronology is derived by using all available ring-width series from the four sub-chronologies. The thick line is the 11-year Fast Fourier Transform (FFT) smoothed series. To the right of the vertical dotted line the chronology EPS value is above 0.85, which indicates a statistical reliable series. The light yellow shaded area shows the number of series. The numbers in the upper left corner of all subfigures are the elevation ranges of each zone.

**Figure 8:**
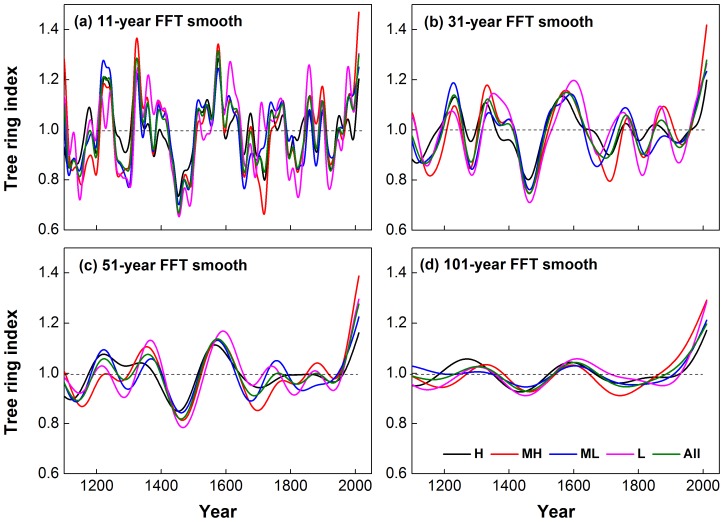
Comparison of low-frequency variability in four STD chronologies at different elevations and the composite chronology during 1110–2011.

Four STD and RES chronologies were established at four elevations (Figure 7). The mean sensitivity and first-order autocorrelation of the tree ring indices are considerably reduced relative to the values for ring width data. However, the percent variance explained by the first principal component of the index data is increased by comparison. The high correlation coefficients between all the chronologies (listed in Table 2) show that there are no significant statistical differences between standardized tree ring chronologies at different altitudes for the periods 1110–2011 (*R>0.81, N = 902, p = 0.01*) and 1956–2011 (*R>0.88, N = 56, p = 0.01*). It is worth noting that RES showed better correlation than STD at the four elevations, due to removal of more of the lower order trends. That is to say, there is greater consistency in the high-frequency variability than in medium or lower-frequency trends, though there were no statistically significant differences apparent in the lower-frequency trends in the chronologies. The consistency between the STD chronologies is shown by Fast Fourier Transform (FFT) filter smoothing of the chronologies at different time scales during the last 902 years (Figure 8). Principal components analysis of these data shows that the first component (PC1) explains 91.3% variance, with very similar loadings of 0.97/0.97/0.94/0.94 corresponding to the H/MH/ML/L chronologies respectively. Therefore, all four chronologies from different elevations exhibit a very strong common pattern of variability during the past 902 years. Finally, STD and RES chronologies were developed using all increment cores collected from the four altitude belts, and is referred to here as “All” chronology (Tabs. 1–5, 8; Figure 8).

**Table 2:** Correlation coefficient matrix between the four STD (upper right corner) and RES (lower left corner) chronologies and the composite chronology during 1956–2011 and 1110–2011.

R	H	MH	ML	L	All
H	1	0.91/0.92	0.88/0.87	0.88/0.81	0.96/0.95
MH	0.95/0.94	1	0.94/0.92	0.89/0.88	0.97/0.98
ML	0.92/0.91	0.92/0.95	1	0.89/0.90	0.96/0.96
L	0.93/0.87	0.93/0.93	0.9/0.93	1	0.95/0.92
All	0.98/0.97	0.98/0.99	0.95/0.97	0.97/0.95	1

All the values are significant at the p = 0.01 level.

**Table 3:** Comparisons of the three optimum seasonal assemblies of various climate elements for each of the STD chronologies.

	Temperature		Min.Tem.		Max.Tem.		Precipitation		PDSI	
	SA	R	SA	R	SA	R	SA	R	SA	R
	P9-C3	0.24	P9-C3	0.19	P9-C3	0.26	P7-C6	0.6	C3-C6	0.5
H	P1-P12	0.23	P1-P12	0.19	P1-P12	0.21	P12-C6	0.56	P9-C8	0.46
	P12-C2	0.24	P12-C2	0.2	P12-C2	0.26	P3-C6	0.56	P9-C2	0.33
	P9-C3	0.51	P9-C3	0.48	P9-C3	0.38	P7-C6	0.74	C3-C6	0.57
MH	P1-P12	0.49	P1-P12	0.48	P1-P12	0.3	P12-C6	0.67	P9-C8	0.48
	P12-C2	0.46	P12-C2	0.45	P12-C2	0.37	P3-C6	0.65	P9-C2	0.31
	P9-C3	0.46	P9-C3	0.45	P9-C3	0.28	P7-C6	0.68	C3-C6	0.61
ML	P1-P12	0.45	P1-P12	0.45	P1-P12	0.25	P12-C6	0.66	P9-C8	0.51
	P12-C2	0.41	P12-C2	0.43	P12-C2	0.25	P3-C6	0.65	P9-C2	0.34
	P9-C3	0.28	P9-C3	0.29	P9-C3	0.11	P7-C6	0.61	C3-C6	0.46
L	P1-P12	0.28	P1-P12	0.3	P1-P12	0.09	P12-C6	0.58	P9-C8	0.4
	P12-C2	0.25	P12-C2	0.27	P12-C2	0.13	P3-C6	0.58	P9-C2	0.27
	P9-C3	0.39	P9-C3	0.37	P9-C3	0.26	P7-C6	0.69	C3-C6	0.55
PC1	P1-P12	0.38	P1-P12	0.37	P1-P12	0.22	P12-C6	0.65	P9-C8	0.48
	P12-C2	0.35	P12-C2	0.35	P12-C2	0.26	P3-C6	0.64	P9-C2	0.32
	P9-C3	0.38	P9-C3	0.36	P9-C3	0.26	P7-C6	0.68	C3-C6	0.56
All	P1-P12	0.37	P1-P12	0.36	P1-P12	0.21	P12-C6	0.64	P9-C8	0.48
	P12-C2	0.34	P12-C2	0.34	P12-C2	0.26	P3-C6	0.63	P9-C2	0.32

Min. Tem.: monthly minimum temperature; Max. Tem.: monthly maximum temperature; PDSI: Palmer Drought Severity Index; SA: optimum seasonal assembly; R: correlation coefficient; H/MH/ML/L: the site code of the four different elevations (see Table 1); PC1: first principal component of the four chronologies; All: the composite chronology; P9-C3: previous September to Current March; 0.24/0.11: correlation coefficient with STD and RES chronologies, respectively.

**Table 4:** Pearson correlation coefficients (1956–2011) between STD chronologies and optimal seasonal precipitaiton and temperature and the same correlations using first-order differential series.

	Precipitation(P7-C6)		Temperature (P12-C2)	
	R	R_ac1_	R	R_ac1_
H	0.6	0.62	0.24	0.04
MH	0.74	0.69	0.46	0.02
ML	0.68	0.62	0.41	−0.03
L	0.61	0.66	0.25	−0.06
All	0.68	0.66	0.34	−0.01

R: correlation coefficient; **R_ac1_**: correlation coefficient of the first-order differential series. H, MH, ML, L, All are site codes for different elevations (see Table 1). Precipitation (P7-C6) is the accumulated precipitation from previous July to current June. Temperature (P12-C2) is the mean temperature from previous December to current February.

**Table 5:** Response surface regression results and their F values at the four different elevations and the composite site.

Site	A_00_	A_01_	A_02_	A_10_	A_11_	A_20_	F	F_0.01_(5,49)/F_0.01_(5,48)
H	0.142/−0.1	−0.264/0.163	−0.216/0.149	0.866/0.6	0.573/0.022	−0.259/−0.047	8.8/7.4**	3.42/3.43
MH	0.116/−0.081	0.025/0.189	−0.12/0.129	0.863/0.67	0.496/0.022	−0.284/−0.046	19.1/10.7**	3.42/3.43
ML	0.128/−0.025	0.018/0.156	−0.121/0.097	0.761/0.608	0.315/0.004	−0.19/−0.071	10.2/7.3**	3.42/3.43
L	0.328/−0.115	−0.278/0.12	−0.227/0.163	0.917/0.626	0.351/0.081	−0.309/−0.045	8.9/9.3**	3.42/3.43
All	0.199/−0.09	−0.148/0.16	−0.181/0.141	0.901/0.638	0.461/0.037	−0.287/−0.049	11.8/9.2**	3.42/3.43

The response surface was defined by a quadratic surface function: 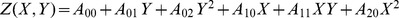.

Here, Z = Z score value of standardized tree ring index at different elevations and the whole site; X = Z score value of mean precipitation (previous July to current June); Y = Z score value of mean temperature (previous September to current March). The F value is calculated by 
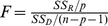
, with notation as follows. 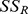: regression sum of squares, 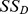: residual sum of squares, *p*: degrees of freedom of the regression function, here *p* = 5, n: number of active data, here n = 55. If 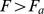, the response surface regression is significant at the α level.

** F value is significant at p = 0.01 level.

A common period analysis for the regional chronology was carried out for the period 1750–2000, and some descriptive statistics are shown in Table 1. Signal strength of the standard chronology was assessed by the mean inter-series correlation (rbar), and the associated expressed population signal (EPS). The Rbar (>0.4), and EPS (>0.90) during 1750–2000 indicate that signal strength was sufficient for climate reconstruction of the medium to high-frequency climate variability.

### Growth-climate response

**Figure 9:**
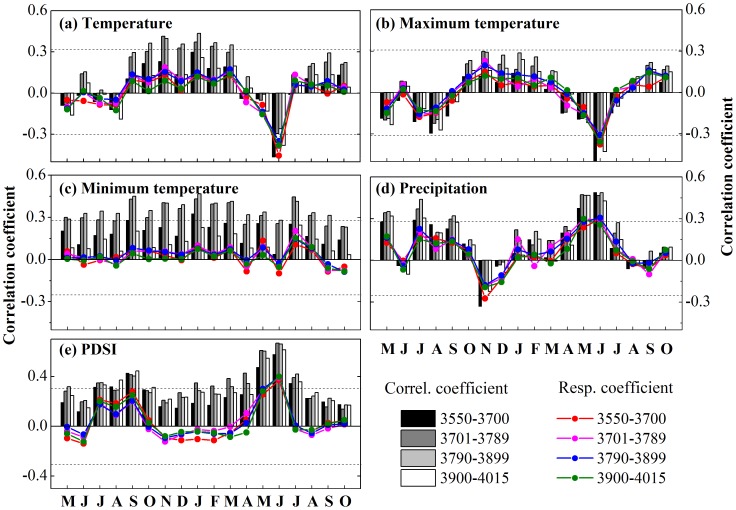
Correlation (histogram) and response function (line) between the four STD chronologies at different elevations and monthly mean values of temperature (a), maximum temperature (b), minimum temperature (c), precipitation (d), and PDSI (e). The horizontal dashed line indicates the 95% confidence level for the correlation.

Figure 9 shows that, as expected, there is a similar pattern in single-month correlation function analysis between climate variability and the different elevation STD chronologies over the common period 1956–2011. Correlation with TRW and mean temperature is significantly positively correlated during the winter half year (from previous September to current March) and is significantly negatively correlated with temperature during the current June. However, only the correlation with temperature in the current June is confirmed to be significant (at the 0.05 level) by the response function analyses. The relationship between maximum temperature and TRW was similar in general, except for slight differences in the magnitudes of changes. There was a different pattern in the minimum temperature, which appears to be positively correlated in every month. However, this was not verified by the response function analysis. Apparently significant positive correlations were found for monthly precipitation in May, July and September of the previous year and May and June of the current year, while a significant negative correlation was found for November of the previous year. Correlation with precipitation in the current May and June was confirmed by response function analysis, which is also validated by the process-based Vaganov-Shashkin models [42] in this region. The correlations between TRW and PDSI are positive in every month, as confirmed for the current May and June in the response function analysis.

**Figure 10:**
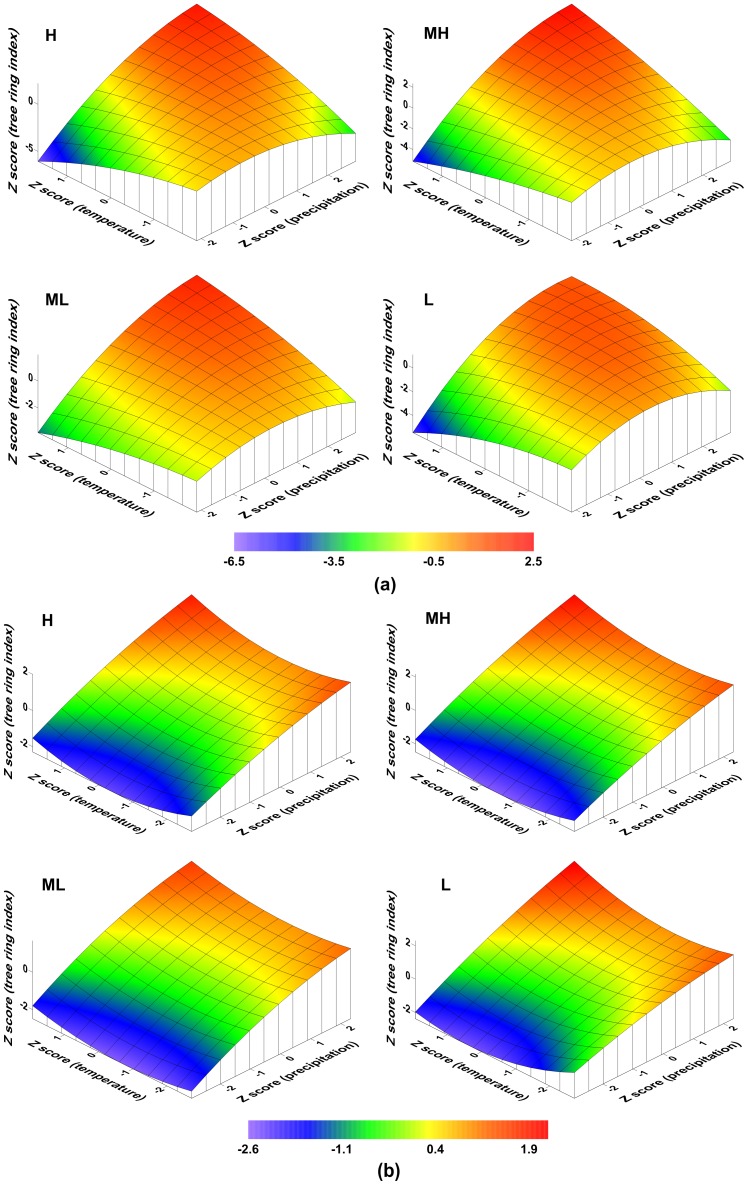
Response surfaces for standardized tree ring index (a) and first-order differential series of standardized tree ring index (b) for four different elevations: H, MH, ML, L. The height of each surface represents the magnitude of growth response to a given combination of annual (previous July to current June) precipitation and winter (previous September to current March) temperature. All data (including precipitation, temperature, and standardized tree ring index) are Z score transformed in order to eliminate the impact of different dimensional units.

Table 3 shows the apparent optimum seasonal associations are P7-C6, P9-C3, and C3-C6 for precipitation, temperature and PDSI, respectively. Here, we focus attention on precipitation and temperature because these strongly influence PDSI. We first focus on the winter temperature and compute the Pearson's correlation coefficient between STD chronologies and winter temperature along with its first-order differential series at different elevations (Table 4). This reveals that there is no correlation (R = −0.06–0.04, N = 56) on inter-annual timescales (i.e. with the high frequency signal) and that the raw correlation is based only on a correspondence in trend (R = 0.24–0.46, N = 56). Unfortunately, the longer-term climate association cannot be further explored with only 56 years of instrumental records. However, there is a distinct pattern between precipitation and tree radial growth (Table 4). There is a strongly positive correlation (R = 0.62–0.69, N = 56) between precipitation and tree growth on inter-annual time scales. This is more evident for the climate response surfaces calculated using the STD tree ring index (Figure 10a, Table 5) and its first-order differential series (Figure 10b, Table 5) in the four different elevations. These results lead us to conclude that any temperature-related inference based on these chronologies relies solely on a statistical association manifested only at low-frequency and it cannot be considered more than a coincident association in recent decades at these sites. Tree rings best represent precipitation variability, as might be expected given the severe lack of water at these sites.

### Elevation effects on the chronology variability

**Figure 11:**
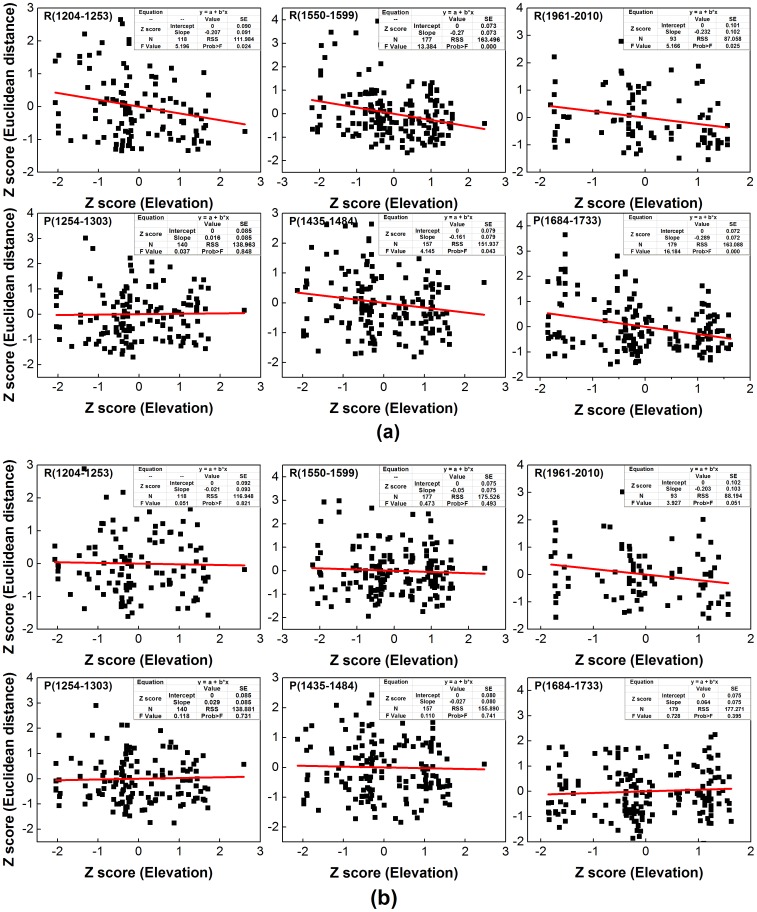
Comparison of altitude-related effects on standardized tree ring series (a) and residual tree ring series (b), which separately constitute the STD and RES chronologies during six typical periods (three high-value periods and three low-value periods). Euclidean distance is defined as the distance between each TRW series (50-dimensional vectors) and the mean TRW series (50-dimensional vector) of all the TRW series in the given periods. Both the elevation (m) and Euclidean distance are transformed to Z scores. R(1204–1253): high precipitation during 1204–1254; P(1254–1303):low precipitation during 1254–1303; SE: standard error; RSS: residual sum of squares; Prob>F: the probability that the null hypothesis for the full model is true (i.e., that all of the regression coefficients are zero).

**Figure 12:**
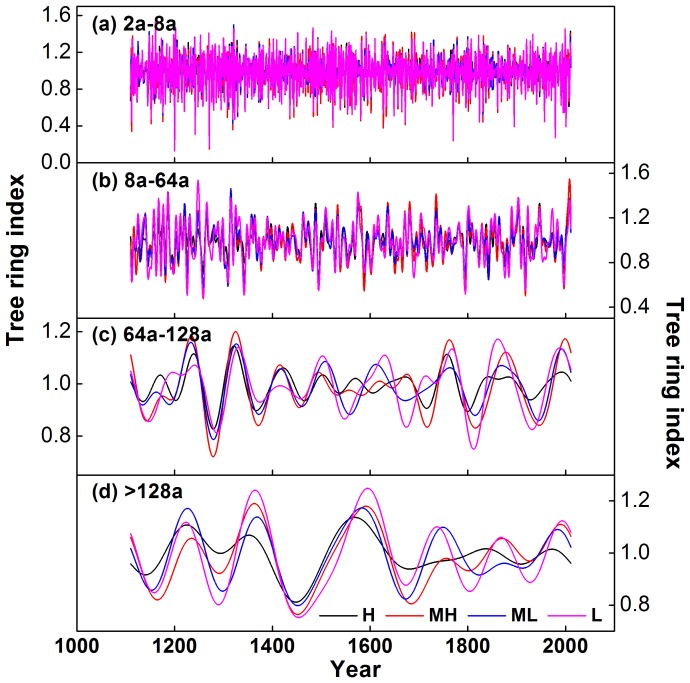
Comparisons of different frequency components of STD chronologies at four elevations during 1110–2011. The four frequency components are 1/8 to 1/2 cycles per year (cpy) (a), 1/64 to 1/8 cpy (b), 1/128 to 1/64 cpy (c), and less than 1/128 cpy (d), respectively.

To examine the natural role of altitude-related effects on chronology variability during different ‘typical’ periods (Table 6) in the last 902 years, we calculated the Euclidean distance for each of the tree-ring index series (i.e. for each single tree) in both the STD and residual data sets during each period. We first examined the altitude-related characteristics for both the mean tree biological age and sample size (Table 6) in order to assess whether a heterogeneous sample distribution might contribute to the tree biological age curve, or whether different sample sizes occur at different elevations. Most of the six periods have a relative even altitude distribution with respect to both the mean tree biological age and sample size, except for 1254–1303 and 1961–2010. So we considered the remaining four periods would most likely indicate any true altitude-related effect. Significant negative correlations ranging from −0.29 to −0.16 (with p values ranging from about 0 to 0.04, Figure 11a) were obtained for the relationships between elevation and standardized tree-ring series during most periods (the exception was 1254–1303: correlation r = 0.02, p = 0.85), which is anomalous with respect to the analysis results for the four chronologies at different elevations. Various “individual responses” of TRW index [26], [43] are the most likely potential causes of such contradictory conclusions. However, there were no significant negative correlations with altitude in the residual tree ring series for any of the periods (Figure 11b) (correlation range r = −0.05 to 0.06, p = 0.40 to 0.82) with the exception of 1961–2010 (r = −0.20, P = 0.05). Therefore, there is an apparent altitude-related effect on standard tree ring index series, and no statistically discernable effect in the residual tree ring series. This suggests some systematic tendancy for greater growth at lower elevation associated with the greater low-frequency variability presented in the indices produced for standard chronologies. This finding is also confirmed using other methods for growth trend removal (e.g. Hugershoff; not shown in this article). It should be noted that while such effects are statistically significant on the scale of a single TRW index series level, they cannot be diagnosed using the four STD chronologies (i.e. after averaging individual trees index series). From the inter-annual, decadal, centennial to multi-centennial scale, there are no notable differences in the STD chronologies for the four elevations from 1110 to 2011 (Figure 12, Table 7). This finding is confirmed by the tree-ring study conducted at Qifeng site (∼235 km far away) in the northeast Tibetan Plateau [13]. However, this situation contrasts with the view that climate responses are frequency dependent: e.g. where low-frequency variations from the upper forest border chronologies are likely related to warm-season temperature fluctuations, whereas high-frequency variations are related to precipitation, such as in the semiarid mountainous area of North America at similar latitudes [44], [45]. Such differences in the frequency-dependent climate signal are due to different climate responses for the upper (temperature) and lower (precipitation) border chronologies, and may not be applicable to a region such as this or where the climate responses at the upper and lower forest borders are unknown.

**Table 6:** Comparisons of altitude-related characteristics for the mean tree biological age and corresponding sample size in six typical periods.

Elevation/m	1204–1253/AD	1254–1303/AD	1435–1484/AD	1550–1599/AD	1684–1733/AD	1961–2010/AD
3551–3600	63/6	104/9	229/10	338/11	284/15	265/12
3601–3650	69/1	119/1	51/3	155/4	73/15	355/3
3651–3700	176/9	217/10	369/11	490/10	582/4	183/4
3701–3750	108/10	156/11	146/13	232/14	305/15	576/8
3751–3800	108/36	155/41	236/43	270/43	249/51	351/27
3801–3850	284/11	245/13	186/10	274/12	351/11	417/5
3851–3900	55/12	93/16	241/19	260/25	215/14	517/7
3901–3950	78/14	121/16	254/19	303/24	199/16	407/8
3951–4000	113/12	111/16	233/21	284/25	153/33	380/16
4001–4050	149/6	199/6	274/7	288/8	312/5	589/3
>4050	150/1	150/1	150/1	150/1	150/1	

**Table 7:** Comparisons of correlations of different frequency components for four elevation STD chronologies.

R*	H	MH	ML	L
H	1	0.96/0.92	0.93/0.85	0.91/0.75
MH	0.84/0.76	1	0.96/0.92	0.96/0.83
ML	0.74/0.78	0.87/0.82	1	0.95/0.87
L	0.56/0.68	0.70/0.86	0.81/0.86	1

* 1/8 to 1/2 cpy and 1/64 to 1/8 cpy are on the upper right corner; 1/128 to 1/64 cpy, and less than 1/128 cpy are on the lower left corner.

All the values are significant at the p = 0.01 level.

**Table 8:** Kruskal-Wallis H test on different missing ring ratio events during 1200–2011.

MRR(%)	N	Mean Rank				Chi-Square	Asymp.Sig.(2-tailed)
		H	MH	ML	L		
>10	13	34.77	28	18.46	24.77	7.826	0.05*
10-2	31	58.03	59.27	57.98	74.71	4.847	0.183
<2	105	208.6	236.82	202.18	194.4	10.435	0.015*
All	149	293.11	315.06	289.29	296.54	2.29	0.515

MRR: missing ring ratio; N: number of events; Mean Rank of H/MH/ML/L: mean ranks within each altitude belt; Chi-Square: Chi-Square coefficient; Asymp. Sig.: asymptotic significance, which means that the significance is very close to 0.

* the value is significant at the p = 0.05 level.

### Extreme climate events and their potential as climatic controls on tree growth

**Figure 13:**
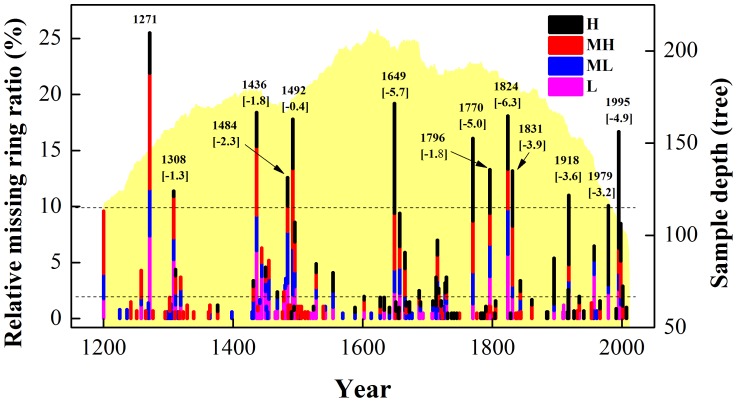
Comparison of relative missing ring ratios at four altitude belts and for the whole hillside in MNT, Delingha, northeast Tibetan Plateau during 1200-2011. Sample depth homogenization was applied to reduce sample-depth-related disturbance prior to computing the relative missing ring ratio. Bar heights indicate missing ring ratios for all samples each year, as visualized by different colored bars (the length of each bar corresponds to the relative missing ring ratio at each elevation). The light yellow shadow is the sample depth. The two dotted lines correspond to the relative missing ring ratios of 10% and 2%, respectively. The values on each bar are the corresponding year and PDSI value (in square brackets, Cook et al., 2010) for those years when the relative missing ring ratio exceeds 10%.

Relative missing ring ratios, at four altitude belts and for the whole hillside in MNT during 1200–2011 are plotted in Figure 13. In order to learn more about whether there are significant differences in missing ring responses to extreme climate variability at different altitudes, we used the Kruskal-Wallis H test. The results (Table 8) show that significant differences exist between high (*>10%, n = 13, p = 0.05*) and low (*<2%, n = 105, p = 0.015<0.05*) missing ring ratio events, although there was no significant difference (*n = 149, p = 0.397>0.05*) between the complete distributions of all the missing ring ratio events across the four altitude belts. As seen from Table 8, the trees near the upper treeline (mean rank H: 208.6, MH: 236.82) are more sensitive to climate change than those in the other altitude belts (mean rank ML: 202.18, L: 194.4), based on a frequency of relative missing ring ratio events of less than 2%. A similar interpretation applies to the data on extreme climate events (relative missing ring ratio greater than 10%, mean rank H: 34.77, L: 24.77). Our analysis suggests that researchers can reduce field work while still gaining valuable insights into the occurrence of past extreme climate events by focusing sample collection at the upper treeline.

**Figure 14:**
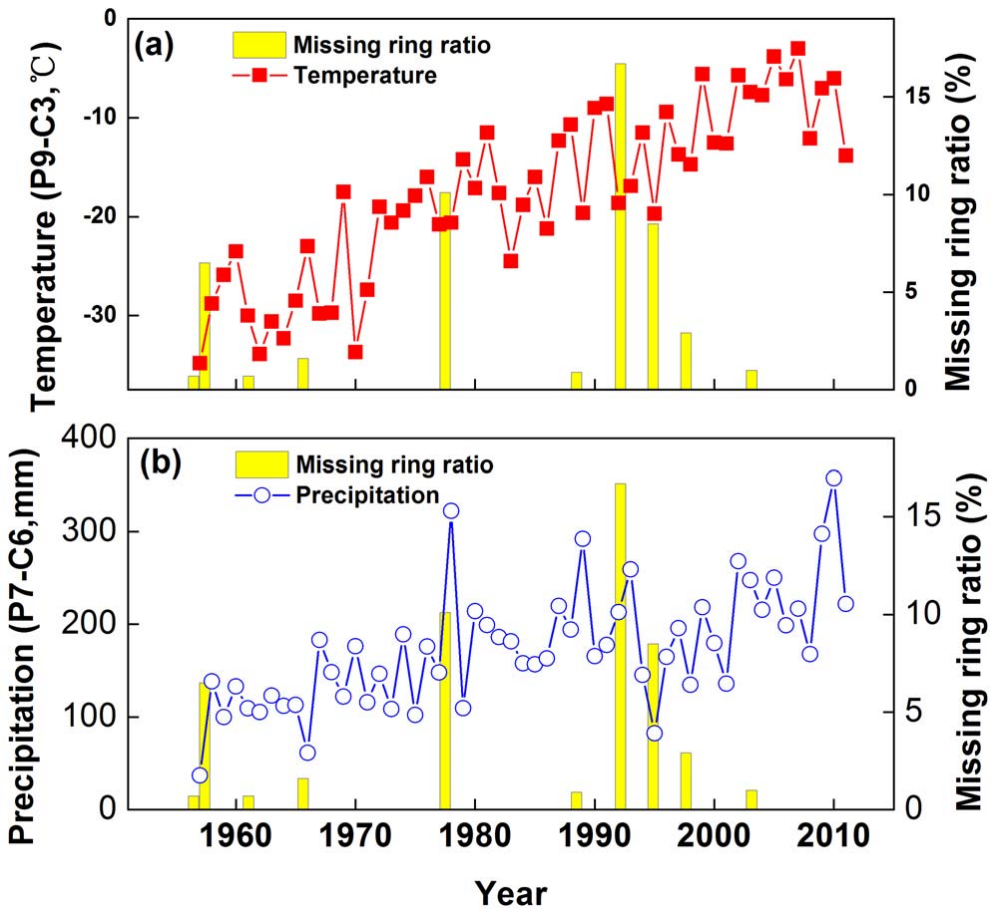
Comparison between missing ring ratio and the temperature (a) and precipitation (b) instrumental records at Delingha during 1956–2011. Temperature is the mean from previous September to current March. Precipitation is the total from previous July to current June.

**Figure 15:**
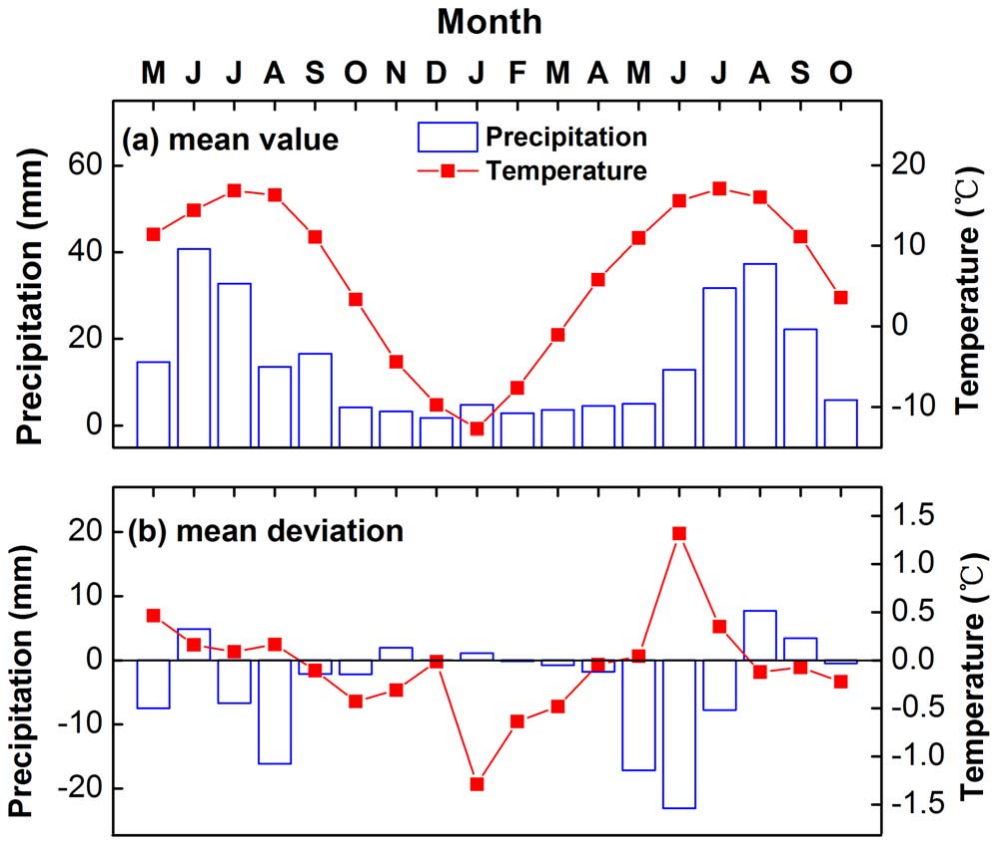
Average (a) and mean deviations (b) of precipitation and temperature from their long-term (1956–2011) mean values for all the missing ring events of the composite chronology.

What is the climatic cause of such missing ring events? Comparing their occurrence (i.e. years with more than 10% missing rings) with reconstructed PDSI data (Cook et al. [46], data for grid location 36.25°N, 98.75°E), reveals a mean PDSI value of −3.3 i.e. extreme dry years (Figure 13). This is confirmed by primary instrumental records during 1956–2011. Compared with temperature, precipitation displays a more robust and consistent relationship with missing ring events (Figure 14). Therefore, the missing ring events, especially those years with a relative missing ring ratio more than 10%, very likely indicate serious drought events. Figure 15 illustrates the composited climate pattern for the 9 missing ring event years that occurred within 1956–2011. Temperatures are “normal” for the year preceding the missing-ring year. There was some water shortage in the prior August (−16.15 mm), but this is likely not a key factor. However, serious water shortage in May (−17.19 mm) and June (−23.07 mm) in the tree growth year, which corresponds to the start period of radial growth are very likely the major cause. Simultaneously, high temperature (+1.32°C) in June probably reinforces the water stress. Extremely low precipitation, accompanied by abnormally high temperatures at the beginning of the radial growth season, is the likely cause of missing ring events.

## Conclusions

Using samples collected from 331 trees at four elevations (3550 m–4020 m) on one single hillside, including 164 trees with exact pith offset information, we analyzed the characteristics of age-related effects and altitude-related effects on TRW in Qilian juniper on the northeastern Tibetan Plateau. Altitude impacts significantly on radial ring width: growth rates were lower at higher altitudes, regardless of age. Tree-ring width reaches a maximum between the ages 25–45.

There is a strong and significant correlation between the ring-width chronologies at four different altitudes. The chronology-climate associations at different elevations, as would be expected on this basis, show no significant differences, whether expressed as single-month correlations or by the use of response function analysis. Standardised tree growth is associated primarily with precipitation variability during previous July to current June. Despite coincident trends, winter temperatures prior to the growing season, have no influence on the high frequency tree growth signal. In the similarity analysis of standardized and residual tree ring series, some significant altitude-related effects were apparent but only on the scale of individual standardized tree ring series, regardless of whether precipitation was relatively high or low. Elevation showed no significant influence on the variability of mean chronologies during the last 902 years or during any of the selected 50-year periods. Missing ring events are related to drought, and the uppermost treeline is more sensitive to climate change than that of low altitude forest. Serious water shortage in May and June of the growth year, which is strengthened by the high temperature in June of the growth year, are the main conditions needed to initiate missing ring events. There is no doubt that our interpretation of tree-growth/climate relationship in the cold and arid region could be helped by a better mechanistic understanding of tree-growth physiology. A constructive way forward may be to link small scale physiological tree growth models, driven by localised meteorological variables, with large-scale climate models and compare the results with radial growth data in this region.
